# Immunological consequences of strain variation within the *Mycobacterium tuberculosis* complex

**DOI:** 10.1002/eji.201646562

**Published:** 2017-02-24

**Authors:** Leopold D. Tientcheu, Anastasia Koch, Mthawelenga Ndengane, Genevieve Andoseh, Beate Kampmann, Robert J Wilkinson

**Affiliations:** ^1^ Vaccines and Immunity ThemeMedical Research Council Unit, The GambiaBanjulThe Gambia; ^2^ Department of Biochemistry, Faculty of ScienceUniversity of Yaoundé 1YaoundéCameroon; ^3^ Wellcome Centre for Infectious Diseases Research in AfricaInstitute of Infectious Disease and Molecular Medicine and Department of MedicineUniversity of Cape TownObservatoryRepublic of South Africa; ^4^ Department of MedicineImperial CollegeLondonUnited Kingdom; ^5^ The Francis Crick InstituteLondonUnited Kingdom

**Keywords:** Adaptive and innate immunity, Genetic diversity, Host response, *Mycobacterium tuberculosis* complex, Translational implications

## Abstract

In 2015, there were an estimated 10.4 million new cases of tuberculosis (TB) globally, making it one of the leading causes of death due to an infectious disease. TB is caused by members of the *Mycobacterium tuberculosis* complex (MTBC), with human disease resulting from infection by *M. tuberculosis* sensu stricto and *M. africanum*. Recent progress in genotyping techniques, in particular the increasing availability of whole genome sequence data, has revealed previously under appreciated levels of genetic diversity within the MTBC. Several studies have shown that this genetic diversity may translate into differences in TB transmission, clinical manifestations of disease, and host immune responses. This suggests the existence of MTBC genotype‐dependent host–pathogen interactions which may influence the outcome of infection and progression of disease. In this review, we highlight the studies demonstrating differences in innate and adaptive immunological outcomes consequent on MTBC genetic diversity, and discuss how these differences in immune response might influence the development of TB vaccines, diagnostics and new therapies.

## Introduction

**Figure 1:**
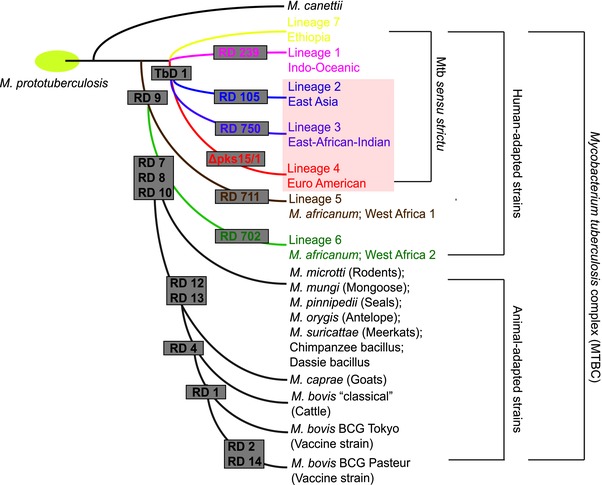
Schematic diagram illustrating the evolutionary relationship between selected members of the MTBC 1, 84, 111, 112. Human‐adapted and animal‐adapted members of the MTBC can be classified according to the absence or presence of deletions known as regions of difference (RD), which were originally defined by 1 Grey boxes indicate the specific deletion event that has occurred on the branches leading to a lineage. Human‐adapted strains of the MTBC are grouped into 7 lineages and are associated with specific geographic regions. In the main text of the article, we refer to these lineages using the numerical nomenclature; however, the alternative nomenclature reflecting the geographic region wherein strains are prevalent is indicated below the numerical name. The presence or absence of the TbD1 regions discriminates modern (highlighted in a pink box) and ancient human‐adapted MTBC strains. Animal‐adapted members of the MTBC, together with the host that is infected by that strain, are indicated on the figure.

Tuberculosis (TB), caused by members of the *Mycobacterium tuberculosis* complex (MTBC), is a chronic disease that affects humans and animals 1. The MTBC includes strictly human pathogens *M. tuberculosis* sensu stricto (Mtb) and *M. africanum* 2 that are classified into different lineages according to their phylogeographic structure, which is hypothesized to reflect adaptation to the human populations in which they cause disease. Animal‐adapted MTBC strains include *M. bovis*, a pathogen of cattle and badgers 3; *M. microti*, found in rodents 4; *M. pinnipedii* infects sea lions 5; *M. caprae* in goats 6; the recently described *M. orygis* which infects wild buck 7 *; M. mungi*, which was isolated in mongoose populations 8 and *M. suricattae* in meerkats (Fig. 1). Zoonotic transmission of MTBC to humans mainly involves *M. bovis*. Other animal adapted members have rarely been isolated from humans, thereby making the humans the only significant natural reservoir responsible for the global TB burden.

With the development of genotyping techniques a phylogeographic genetic structure for the MTBC began to emerge 1 (Table 1). Large insertions and deletions within the genome, referred to as large sequence polymorphisms (LSPs), were initially used to characterize the evolution and phylogeography of the MTBC 1. The association of lineages with specific host populations led to a nomenclature for MTBC lineages which mirrors geographic regions where lineages predominate 9 (Fig. 1). MTBC phylogenies subsequently constructed using whole genome sequence (WGS) data were congruent with those generated using LSPs 10, and are used in this review. Human‐adapted MTBC strains are grouped into seven lineages classified as “ancient” (lineages 1, 5, 6, and 7) or “modern” (2, 3, and 4) according to the presence or absence of the tuberculosis‐specific deletion (TbD1) region (Fig. 1). While modern MTBC lineages (i.e. lineage 2 and 4) have spread globally, most ancient lineages (i.e. lineage 5 and 6 (West Africa) and lineage 7 (Ethiopia)) remain geographically restricted to specific regions/countries where they cause disease 11. Moreover, WGS data has been used to demonstrate significant intra‐lineage diversity in lineage 2 12 and lineage 4 groups 13. In a recent study, Stucki et al. show that certain lineage 4 sublineages are more geographically distributed than others 13.

**Table 1:** Common genotyping techniques for MTBC 111, 113, 114

Technique	Principle	Advantages	Disadvantages
Spoligotyping	• Polymorphism at a direct repeat (DR) locus is used to discriminate strains. The locus is in fact made up of clustered regularly interspaced short palindromic repeats (CRISPRs), and in other bacteria are associated with bacterial immunity to foreign DNA. • PCR and reverse hybridization is applied to detect the presence or absence of 35 – 41bp “spacer” regions, which occur between 36bp repeat sequences.	• Inexpensive • Fast • Minimal DNA required therefore samples do not need to be cultured prior to typing.	• Low resolution • Can't effectively discriminate between MTBC strains, particularly Lineage 2 strains, all of which lack spacers 1 – 34.
IS*6110*‐Restriction fragment length polymorphism (RFLP)	• IS6110 is a 1361bp IS3‐family mobile genetic element which can be found in the varying frequency and loci within the genome. • The number of copies and the site of IS6110 insertion are detected via restriction digest followed by blotting and labelling.	• Discriminatory power is better than spoligotyping and can resolve differences between MTBC lineages.	• Can't be used to accurately type isolates with fewer than 5 IS6110 bands. • Requires large amounts of good quality DNA.
Variable‐number tandem‐repeat typing (VNTR)	• Repetitive regions (40 – 100bp in length) that are found at 41 loci throughout the chromosome, with varying number of repetitive units. The regions are also known as mycobacterial interspersed repetitive units (MIRUs) and therefore the technique can be referred to as MIRU‐VNTR. • Polymorphism is detected via PCR using primers specific for the flanking regions of the repeats. Can be performed on 15‐ or 24‐loci, with 24‐loci providing better resolution.	• Better discriminatory power than both RFLP and spoligotyping. • Minimal DNA required therefore samples do not need to be cultured prior to typing.	• Automation requires a sequencer and specialized software.
Whole genome sequencing (WGS)	• The full genome sequence of the isolate is determined. The most common technique applied is shotgun sequencing, generating the sequence of short reads (25 – 450bp) after fragmentation of the genome. Reads are then aligned to a reference genome. • Newer technologies, such as the Nanopore and PacBio systems, can sequence single molecules of DNA to generate long reads 115. These have not been widely applied to MTBC due to cost and availability of technology.	• Unrivalled resolution and discriminatory power. • Can provide lineage and drug resistance information. • Can provide information about specific SNP differences at specific loci, allowing investigation of the functional implications of genetic diversity.	• Short read sequencing data can't be used to determine the sequence of repetitive regions. This is major disadvantage for Mtb given that ∼10% of the genome comprises repeat regions of which the PE/PPE genes being significant for the proposed role in host‐pathogen interactions. • Expensive • Specialized software and skilled personnel required for data analysis. • Standard techniques require large amounts of good quality DNA.

Differences in virulence between MTBC strains were reported as early as the 1960s, with the observation that MTBC isolated in India were attenuated in the guinea pig model when compared with those isolated in the UK 14. When genotyping techniques became available in the 1990s, outbreaks caused by specific MTBC strains could be delineated and these strains have been characterized in a range of models 15. Two of the most highly chacterized outbreak strains are Mtb CDC1551and HN878. The CDC1551 strain, a member of lineage 4, was responsible for a TB outbreak, that exhibited an unusually high rate of tuberculin skin test (TST) conversion in contacts 16. Subsequently it was shown that CDC1551 induced high levels of IL‐12 and IFN‐γ in mice, which may explain the observation of high levels of TST conversion 17. The HN878 Mtb, a lineage 2 strain, was implicated in multiple outbreaks in Houston, Texas, USA, and was shown to be highly virulent in mice 18 and rabbits 19. The HN878 strain, and most other members of the lineage 2 strain family, produces a phenolic glycolipid that dampens the proinflammatory response, suggesting one mechanism for the increased virulence of these strains 20. In addition to transmission, associations between specific MTBC lineages and the site of disease 21, clinical presentation, and the duration of disease 22 have been observed.

Evidence of significant differences in the transmission and manifestations of disease of MTBC lineages worldwide (reviewed in 23), and variable evidence for host genetic polymorphism and susceptibility to MTBC 24, suggests that genetic variation within the MTBC may influence the host–pathogen interaction and outcome of the infection. In this review, we specifically consider the immunological consequences of MTBC lineage diversity on the innate and adaptive response and discuss the potential translational implications of differences in the immunological response to different MTBC strains.

## The impact of MTBC lineage diversity on the interaction with innate immune cell receptors

Innate immune cell receptors are the first point of contact with MTBC bacilli, and thus play an important role in downstream immune response to MTBC. A wide range of pathogen recognition receptors (PRRs) are involved in the initial interaction between MTBC and host cells, including toll like receptors (TLRs), C‐type lectin receptors (CLRs), nod like receptors (NLRs) and scavenger proteins 25, 26. The cell wall structure of MTBC is complex, including glycolipids and lipoproteins that are uniquely found in pathogenic mycobacterial species and can vary between lineages 27. Together with secreted effector proteins, cell wall components act as pathogen‐associated molecular patterns (PAMPs) that are recognized by host immune cells. Subtle differences in cell wall components can impact the immune response 28 and in addition to activating the immune response, many cell wall components have immunomodulatory functions, which can modulate the immune response in favor of the bacterium 25. In the following sections we highlight experimental evidence that demonstrates variation in PAMPs between MTBC lineages that may influence interaction with PRRs.

### C‐type lectins

C‐type lectins are one important class of receptor that function in innate immunity to TB. Alveolar macrophages highly express Mannose‐receptor (MR, CD206), while dendritic cell specific intercellular‐adhesion‐molecule‐3 grabbing non‐integrin (DC‐SIGN, CD209) is mostly highly expressed on dendritic cells (DC) 29. MTBC cell wall products such as lipoarabinomanan (LAM) and mannosylated‐lipoarabinomanan (Man‐LAM) 29, 30 are recognized by these receptors. Phagocytosis of MTBC via this pathway leads to a reduced inflammatory response thereby presenting an important route of entry from the perspective of the bacillus 27, 30. Structural variation in LAM between virulent and attenuated Mtb laboratory strains results in lower macrophage uptake in vitro of the attenuated Mtb H37Ra strain when compared with more virulent H37Rv or Erdman strains 31. Similar differences have been reported amongst clinical MTBC. A set of lineage 2 isolates were identified as being deficient in uptake by human monocyte‐derived‐macrophages (MDMs) in vitro when compared with the Erdman Mtb strain (a lineage 4 strain) 32. While links with diversity in lipid metabolizing genes were not uncovered, biochemical characterization revealed that ManLAMs in these strains where truncated and more highly branched, leading to reduced surface exposure and thus decreased engagement with the MR 32.

### Toll‐like receptors (TLRS)

Toll‐like receptors (TLRs) are another set of PRRs that are important for sensing MTBC infection 25. Differential recognition of MTBC lineages may impact the response of phagocytes and cytokine production 33. Lipid and protein antigens from MTBC can be recognized by TLR4 and human polymorphism in TLR9 is associated with TB susceptibility; however, due its affinity for lipids, TLR2 is thought to be the dominant TLR for recognition of MTBC 25, 34. Lipid fractions from a lineage 2 isolate, the H37Rv laboratory strain, and *M. canettii* have also been shown to differentially affect TLR2 and TLR4 expression on macrophages. Human MDMs produced higher levels of TNF‐α and IL‐10, and expressed lower levels of TLR2 and TLR4 when stimulated with lipid fractions from a lineage 2 strain when compared with H37Rv and *M. canettii* 35. In a subsequent study characterizing the ability of lineage 2 strains to activate TLR2 and TLR4 receptors in mice, Carmona et al. (2013) show that for most strains studied, cytokine production by macrophages was induced via TLR2 activation 33. However, a specific lineage 2 strain activated the TLR4 receptor on bone marrow‐derived macrophages, leading to production of IFN‐β, increased bacterial burden and lung pathology during the early stages of mouse infection 33. A study in mice demonstrated that the lineage 2 HN878 Mtb strain induced lower levels of mRNA for Th1 cytokines, TNF‐α, IL‐6, IL‐12, and IFN‐γ, and higher levels for mRNA encoding type 1 IFNs, potentially explaining the increased virulence of this strain in mice 18. While this study did not directly address the role of TLRs, preferential activation of TLR4 may partially explain this phenotype. However, Carmona *et al*. (2013) also demonstrated that activation of TLR4 was not specific to lineage 2 strains, with the Mtb Haarlem sub lineage strain, a member of lineage 4, also able to activate TLR4 with concomittant production of IFN‐β 33. In addition, the production of IL‐17 via a TLR2‐dependent pathway was shown to be protective for mice infected with HN878 but not H37Rv 36. These studies demonstrate that immunological differences associated with MTBC strain variation are likely to arise from the interaction between multiple factors and immune receptors.

An interaction between host and bacterial genotype has been observed with respect to TLRs and susceptibility to disseminated TB disease 37 (Table 2). In particular, lineage 2 isolates were more likely to cause meningeal tuberculosis in individuals with the C allele of the T597C polymorphism in TLR2. While the implications for this allele on TLR2 function is not known, this study suggests an important interaction between TLR2 polymorphism and bacterial genotype in TB susceptibility 37.

**Table 2:** Studies describing the impact of both MTBC genotype and host genotype on immunological outcomes of TB infection

Geographic location	Variation in immune component	Methods used to genotype MTBC strains	Key finding	Reference
Ghana	5‐lipoxygenase (*ALOX5*)	• Clinical MTBC strains were genotyped by spoligotyping and IS*6110* fingerprinting	• Heterozygous “5/non 5” promoter mutation that results in lower levels of 5‐LO was associated with TB susceptibility regardless of MTBC lineage. • The exonic variant G760A in males was associated with TB caused by infection by lineage 6 strains.	96
Ghana	Autophagy related human immunity‐related GTPase M (IRGM)	• Clinical MTBC strains that were genotyped by spoligotyping, MIRU‐VNTR, IS*6110* fingerprinting and *pks1‐15* region typing.	• The IRGM genotype –261TT was associated with relative protection against TB caused by *Mtb* lineage 4 but not *M. africanum*. • Stratification of MTBC strains showed that protection was specific against TB caused by lineage 4 strains with a disrupted *pks1‐15* gene.	116
Ghana	Mannose Binding Lectin (MBL)	• Clinical MTBC strains genotyped using LSP typing, IS*6110* fingerprinting and typing of the *pks1‐15*.	• *MBL2* G57E variant was associated with protection against TB caused by *M. africanum* but not Mtb sensu stricto.	42
Ghana	Macrophage Chemoattractant Protein 1 (MCP‐1)	• Clinical MTBC strains genotyped IS*6110* fingerprinting, spoligotyping and MIRU‐VNTR.	• MCP‐1 genotypes variants were associated with resistance to tuberculosis and this was not affected by MTBC lineage differences.	117, 118
The Gambia/ Guinea‐Bissua	Epiregulin (EREG) and V‐ATPase (TCIRG1)	• Clinical MTBC strains genotyed using spoligotyping	• Marginal association between rs11228127 in TCIRG1 and patients infected with *M. africanum* in The Gambia.	119
South Africa	HLA class I	• Clinical MTBC strains genotyped by spoligotyping and IS*6110* fingerprinting.	• Associations between several lineages and specific HLA haplotypes e.g. A*03 was associated with infection by lineage 4 strains.	120
Vietnam	TLR2 and TIRAP	• Clinical MTBC strains genotyped by IS*6110* fingerprinting, large sequence polymorphsm and MIRU‐VNTRs.	• C allele of TLR‐2 T597C was associated with TB disease caused by lineage 2 MTBC strains.	37
Vietnam	Macrophage receptor with collagenous structure (MARCO)	• Clinical MTBC genotyping by IS*6110* fingerprinting, MIRU and *pks1‐15* region typing.	• Two heterozygous (AG) genotypes (rs2278589 and rs6751745) were associated with impaired phagocytosis of MTBC and increased susceptibility to lineage 2 strains but not lineage 4 and lineage 3 Mtb strains.	121
Indonesia	*SLC11A1* gene (also known as *“NRAMP1”*)	• Clinical MTBC strains genotyped by spoligotyping.	• Lineage 2 strains were significantly associated with the G allele and the GG phenotype of the D543N polymorphism compared to non‐lineage 2 strains.	122
Russia	CD209 (DC‐SIGN)	• Clinical MTBC strains genotyped by spoligotyping and MIRU‐VNTR.	• Strong association between the G allele of *CD209 ‐336A/G* and increased mortality from TB caused by lineage 2 strains.	123

### Mannose binding lectin (MBL)

Mannose Binding Lectin (MBL), a member of the collectin family, acts as an opsonin by binding pathogen cell surface carbohydrates 38, 39. While some studies have reported that patients with high serum MBL are more susceptible to TB (reviewed in 40), others suggest the opposite (reviewed in 41). A study conducted in Ghana suggests that diversity between MTBC lineages might partially explain this difference. The study showed that a relatively high proportion of the population studied had an MBL polymorphism 42 that results in lower serum availability of functional MBL by distrupting formation of oligomers 43. The polymorphism was associated with a protective effect against infection with strains of the *M. africanum* lineage but not strains from Mtb sensu stricto lineages. Furthermore, *M. africanum* strains were better able bind to wild‐type MBL than the H37Rv Mtb strain in vitro, suggesting that *M. africanum* uses this receptor as an entry ligand thereby promoting infection. This potentially explains why polymorphisms in MBL are protective against *M. africanum* but not Mtb sensu stricto 42.

These studies highlight the need to take human genetic variation into account when considering differential immunological outcomes associated with MTBC strain variation. Studies investigating association between polymorphism and human susceptibility to TB disease have yielded discrepent results (reviewed in 24, 44), and relatively few studies have taken MTBC genetic diversity into account (highlighted in Table 2). Discrepant associations between polymorphism in host and TB susceptibility might be partially explained by MTBC variation. Matching bacterial and host genotypes presents a challenge to the field. To produce generalizable insights going forward, these studies should involve more than one population and be large enough to be well powered.

## The response of innate immune cells to diverse MTBC lineages

The early response to MTBC infection is characterized by a systemic inflammatory response that is mediated by macrophages, neutrophils, DC, and innate T cells 26, 34. The observation that a proportion of people heavily exposed to MTBC do not develop evidence of sensitization suggests that the innate immune system may completely clear MTBC before infection is established, highlighting the importance of these mechanisms 45. The innate immune response also activates and augments adaptive responses to MTBC infection. MTBC has developed various mechanisms to interfere with effective innate immune function and variation in the capability of various MTBC strains and lineages to do this may significantly influence the outcome of MTBC infection.

### Macrophages

Monocyte and macrophage cell populations have been widely used to study differences in virulence between MTBC lineages. In the following section we highlight some studies that have investigated the macrophage and macrophage‐like cell response to different MTBC lineages.

A study comparing the capacity of MTBC strains, to grow and stimulate cytokine production in primary human monocytes and THP‐1 macrophage‐like cells, that were either transmitted within households or not transmitted within households suggested that transmitted MTBC isolates grew more rapidly than strains that were not transmitted, in both kinds of cells 46. In addition to growth patterns, cytokine production by monocytes infected with different MTBC isolates has been widely studied. Using representative strains for each lineage, Portevin et al. (2011) showed that modern strains (lineage 2, 3, and 4) of MTBC elicited a lower early inflammatory response, as characterized by cytokine production in human peripheral blood MDMs than ancient MTBC strains (lineage 1, 5, and 6) 47. This suggests that modern MTBC strains have adapted to cause more rapid disease progression and therefore transmission 47. In support of this, mice infected with more evolutionary modern sublineages of lineage 2 MTBC strains, isolated in Brazil and Mozambique, had greater lung pathology and lower survival than mice infected with lineage 2 strains that were more evolutionarily ancient 48. MTBC lineages seem to exhibit a lineage‐specific transcriptional pattern in macrophages 49; however, within the modern MTBC group, sublineage‐specific patterns of cytokine production by macrophages have also been observed 50 suggesting that significant intra‐lineage variation also occurs with regards to transcriptional patterns.

Differences in monocyte activation have been shown to be partially responsible for the differences in virulence of the well‐known CDC1551 and HN878 outbreak MTBC strains 51. In particular, human peripheral blood mononuclear cells (PBMC) infected with CDC1551 produced higher levels of pro‐inflammatory cytokines, IL‐1α/β; IL‐1α, and MIP3‐α, indicative of Th1 type immunity and immune protection. In contrast, HN878 stimulated high levels of IL‐4 and IL‐13, which are characteristic of the Th2 type immune response 51. A study published around the same time demonstrated that production of pro‐inflammatory cytokines in murine bone‐marrow‐derived macrophages was inhibited by a phenolic glycolipid produced by HN878. Deletion of *pks1‐15*, the genes encoding the polyketide synthase enzymes that produce this lipid, led to abrogation of this phenotype directly linking a mycobacterial genetic determinant to an immunological phenotype 20. A lineage 3 strain responsible for a large TB outbreak in Leicster, UK, stimulated higher levels of the anti‐inflammatory cytokine IL‐10 and lower pro‐inflammatory IL‐12p40 from human MDMs, suggesting phagocyte deactivation 52. By systematically complementing deletions specific to this MTBC strain, the authors showed that the phenotypic reaction in macrophages was related to the specific deletion of *Rv1519*. However, the precise function of this gene in MTBC is unknown 52.

### Neutrophils

Neutrophils are the first innate cells to migrate to the site of infection and represent the most highly infected phagocytes in the airways of active pulmonary TB patients 53. Moreover, a whole blood neutrophil driven type I interferon transcriptional profile characterizes active from latent TB 54. Studies have shown that TNF‐α‐activated neutrophils can phagocytose and kill MTBC via α‐defensins and proteases rather than ROI and RNI 55, 56. Outbreaks of multidrug resistant (MDR)‐Mtb caused by strains belonging to the Harlem and LAM family of lineage 4 were shown to induce a differential respiratory burst from human neutrophils 57. In particular, apoptosis of neutrophils was more strongly induced by strains of the LAM family, leading to concomitant production of ROI. By contrast the Haarlem family strain, which has been responsible for an ongoing MDR‐TB outbreak in Argentina, induced significantly less neutrophil apoptosis, suggesting that this ability might be one of the reasons for its success 58. This phenotype may be due to differences in the strucutre of α‐glucans present on the cell wall of the Haarlem strain, which promotes non‐apoptotic neutrophil pathways, allowing the bacteria to sequester and replicate 57.

### Dendritic cells

DC make up a large proportion of MTBC‐infected cells, and play an important role in the response to MTBC by transporting antigen to the lymph nodes where it is presented to and activates naïve T cells 26, 45. The function of DC can be inhibited by MTBC and murine bone‐marrow‐derived DC stimulated with soluble extracts from the HN878 lineage 2 Mtb strain expressed lower levels of surface MHC class II molecules, and led to less activation of CD4^+^ T cells than less virulent H37Rv and BCG strains 59. In a separate study, exposure of human monocyte‐derived DCs to clinical Mtb isolates from India, whose genotypes were not defined, resulted in reduced migration of DCs towards a chemokine ligand important to guide DCs to lymphoid tissue, CCL21, compared to LPS stimulation of DCs 60. A study profiling a relatively large number of clinical Mtb isolates using semi‐global protein arrays and ELISA‐arrays showed that human monocyte‐derived DCs infected with lineage 2 Mtb isolates generally produced lower levels of TNF, IL‐6 and IL‐10 than the H37Rv strain, suggesting that this might be a mechanism of increased virulence of these strains 61. Contrasting results were found in a separate study investigating the virulence of different MTBC strains isolated from TB meningitis patients. In this study, there was no difference in cytokine production by murine bone‐marrow‐derived DC in vitro; however, lineage 2 and 3 isolates displayed higher virulence in mice than lineage 4 strains, with more rapid growth and increased dissemination to the blood and lung pathology 62.

## Variation in the adaptive immune response to MTBC lineages

There is a considerable delay in the onset of the adaptive immune response to MTBC, which primarily consists of a T helper 1 (Th1) T cell response. CD4^+^ T cells differentiate into Th1 cells producing IFN‐γ, TNF‐α and GM‐CSF to activate infected macrophages. These macrophages in turn restrict intracellular MTBC by initiating the formation of RNI and ROI, and by autophagy 63. Th1 cells also produce IL‐2 that promotes proliferation and increased cytokine synthesis by T cells and NK cells and LT‐α (lymphotoxin‐α), which is necessary for recruitment and activation of neutrophils. CD8^+^ T cells also have a role in protection against MTBC. Production of granulysin and serine esterase containing granzymes may directly kill MTBC 64, while the production of cytokines such as IFN‐γ and TNF‐α activates phagocytes, and the production of IL‐2 promotes the proliferation of NK and T cells, indirectly contributing to the control of MTBC 65. While the T cell response has been widely studied, less is known about variation in the B‐cell response in TB. However, recent work characterizing antibodies from humans with active and latent TB suggests that B cells may play a significant role in protection against TB 66, 67. Ultimately adaptive immunity results in the formation of a granuloma comprised of uninfected macrophages that confine infected macrophages, epithelioid cells and neutrophils. This barrier is then surrounded by T and B cells, thus providing a physical barrier to contain MTBC 45.

### T‐cell‐mediated immunity

Characterization of the adaptive response to whole cell MTBC has revealed differences in the T cell response to different lineages. A low dose aerosol guinea pig model of infection was applied to examine differences in virulence of a set of lineage 2 MTBC isolates selected for their differences in transmission 68. All strains elicited an appreciable Th1 response, with increased mRNA levels for IFN‐γ, IL‐12p40, and TNF‐α in the lungs, and the presence of activated T cells by day 30 of the infection. However, two strains, which caused a significantly greater number of secondary cases, elicited higher levels of Foxp3 and TGF‐β mRNA in the lungs at day 60, coinciding with a drop in the number of activated CD4^+^ T cells, suggesting expansion of regulatory T cells 68. A study applied a mouse model to extenstively characterize the virulence and T cell response to the K strain, a lineage 2 Mtb isolate responsible for a TB outbreak in a Korean school 69. The K strain was more virulent in mouse lungs, with rapid growth and more extensive pathology, when compared the H37Rv or H37Ra laboratory strains. In addition to differences in the innate response in the mouse lung, the study demonstrated differences in the kinetics of T cell subtypes in animals infected with different MTBC strains. In the lungs of mice infected with all strains, Th1 T‐bet expressing CD4^+^ T cells had accumulated by 14 days, however, at 28 days, these cells began to decrease in the lungs of mice infected with the K strain compared to the H37Rv and H37Ra laboratory strains. Moreover, CD4^+^ T cells expressing RORγt, thought to represent a Th17 subset, were not present in the lungs of mice infected with the K strain while they were present in appreciable numbers in mice infected with laboratory strains, H37Rv and H37Ra. Similarly to the guinea pig study discussed above, higher numbers of CD4^+^CD25^+^Foxp3^+^CD223^+^IL‐10^+^ T‐regulatory (Treg) cells were observed in K Mtb strain infected mice when compared with H37Rv or H37Ra strains 69. In a separate study, in mice infected with HN878, higher numbers of Treg cells were observed within 21 days of infection when compared with H37Rv, suggesting a potential contribution of this cell type to the virulence of this strain 70. While the genetic determinants driving the mechanisms for these observations are unclear, one possible explanation is that some Mtb strains express yet to be discovered antigens that specifically stimulate the expansion of regulatory T cells 69.

### Epitope variation in MTBC

Unlike many bacterial pathogens, several genomics studies have revealed that MTBC T cell epitopes appear to be genetically conserved 10, 71. However, almost all of the WGS data generated thus far utilizes short‐read sequencing platforms, which are not able to efficiently determine sequence in highly repetitive genetic regions, some of which are antigenic 72. One of the most variable regions of the MTBC genome – the PE/PPE gene family – comprises highly repetitive sequences that are routinely filtered out of WGS datasets. Several of the PE proteins have been shown to elicit both cellular and humoral responses in a wide range of models studied 73. This observation has led to the hypothesis that these regions may be a source of antigenic variation as yet undetected by WGS. However, a study characterizing the immunogenicity of a relatively large set of PE proteins in cattle infected with *M. bovis*, or PBMC from humans infected with Mtb, revealed that both the proportion of responders and the breadth of response, as measured by IFN‐γ production, were positively correlated with sequence conservation 74. The study did not characterize the highly variable PE_PGRS subfamily of these genes; however, computational prediction of epitopes in the PE_PGRS subfamily indicated that epitope encoding regions mostly occurred in conserved PE domain of these proteins 71. Furthermore, the impact of non‐synonymous SNPs occurring in these regions in 94 clinical Mtb isolates was found to be negligible for predicted HLA class I and II binding, and epitope prediction 71. While these findings suggest that epitopes are conserved in MTBC even in genomic regions thought to be variable, a small subset of MTBC epitopes that are highly variable were recently uncovered by comparative genomics 75. The immunogenicity of a selection of these epitopes was determined by measuring IFN‐γ responses in whole blood from HIV‐uninfected TB patients in the Gambia, revealing differential responses to ancestral and variant epitope peptides 75. These findings have important potentially implications for vaccine development, discussed in more detail below.

## Translational implications of MTBC lineage associated variation in host immunity

### Implications of MTBC lineage variation for vaccine development

The translational implications of MTBC lineage variation is perhaps most apparent for the design of effective new TB vaccines. Escape from BCG vaccination is one of the factors proposed to have driven the wide geographic distribution of the lineage 2 genotype 76, 77. A recent reconstruction of the evolutionary history of Mtb lineage 2 strains, by genetic characterization of more than one thousand isolates, suggests that BCG vaccination had minimal impact on the population size of lineage 2, suggesting vaccination was insufficient to prevent lineage 2 dissemination 12. To determine whether this is specific to the lineage 2 genotype or due to general variability in BCG efficacy would require similar characterization of MTBC lineages. Data from BALB/c mice and a rabbit model suggest that BCG vaccination provides lower levels of protection against challenge with lineage 2 isolates 78, 79; however, these findings were not reproduced in C57BL/6 mice 80. A subsequent study conducted in C57BL/6 mice and guinea pigs suggested that BCG conferred acceptable levels of protection to lineage 2 strains isolated in South Africa, but not against those isolated in the USA 81. The authors suggest that variable BCG protection is a function of strain fitness, rather than lineage diversity; however, in all of these studies only a few isolates of lineage 2 strain were investigated 81.

In terms of new vaccines, the recombinant BCG candidate (VPM1002) expressing listeriolysin but defective in urease production, was shown to confer better protection than standard BCG in BALB/c mice, hypothesized as a result of increased antigen translocation into the cytoplasm of infected macrophages and resultant antigen cross‐priming 82. Moreover, this vaccine conferred a similar level of protection against a lineage 2 strain as H37Rv 82. VPM1002 is currently in Phase II trials as a BCG replacement candidate vaccine in HIV‐infected infants, and is proposed for Phase III efficacy trials 83.

As previously discussed, several comparative genomics studies have shown that in MTBC, unlike other bacteria such as *Streptococcus pneumoniae*, T cell epitopes are evolutionarily conserved 10, 75. While the biological consequences are not completely understood, these findings imply that these epitopes are conserved because they fulfill an important biological role, and that immune recognition of MTBC may favor the pathogen 10, 84. One of the proteins in a polypeptide candidate TB vaccine, M72/AS0E1, which recently yielded acceptable safety results in a phase II trial 85, contains a sequence from a PE protein discussed in previous sections. High sequence variability among MTBC clinical isolates has been observed in these specific proteins 86, 87. Although the direct effects of the observed sequence variability on the proteins’ immunogenicity has not yet been established, these findings may warrant further investigation.

### Impact of MTBC lineage diversity on TB diagnostics

Currently available tools for detection of MTBC infection rely on immunological sensitization and thus could theoretically be influenced by MTBC lineage associated immunological variation. The TST has traditionally been used detect latent TB infection (LTBI). The test, which requires subcutaneous injection of purified protein derivative (PPD) cannot differentiate MTBC infection from BCG vaccination or exposure to environmental non‐tuberculous mycobacteria (NTMs), and thus may yield false positive results 88. IFN‐γ release assays (IGRA) are a set of newer generation tests that measure in vitro IFN‐γ production in whole blood or PBMC in response to MTBC antigen stimulation, such as CFP10 and ESAT‐6 (and p38‐55 of Rv2654c) 89. Although IGRA are still unable to discriminate active from latent infection, the absence of the CFP10 and ESAT‐6 in BCG and most NTMs confers on IGRA increased specificity to detect MTBC sensitization 89. However, the attenuated T cell response to ESAT‐6 stimulation in *M. africanum* infected individuals has been shown to affect the interpretation of IGRA results 90. In addition, a number of studies have reported that activated T cell markers 91 and soluble cytokines 92 can be used as reliable biomarker for monitoring the anti‐TB treatment response. It has been shown that the production of 27 proinflammatory and regulatory cytokines in response to ESAT‐6/CFP‐10 stimulation was not significantly different between *M. africanum* and Mtb‐infected patients pre‐treatment. However, the kinetics of production of these cytokines following treatment was significantly different between the two groups, with IFN‐γ and GM‐CSF significantly higher in Mtb compared with *M. africanum* infected patients post‐treatment 93. These studies reveal that the interpretation of cytokine production‐based diagnostic tests for monitoring anti‐TB treatment response may need to account for MTBC lineage variation in West Africa where *M. africanum* is prevalent.

### The influence of MTBC lineage diversity on novel host‐directed therapies

MTBC lineage diversity may also constitute an important factor to consider in the development of host‐directed therapies (HDT). Some HDT aim to augment the host defense against TB by modulating host pathways involved in the elimination of MTBC 94. Due to both host and MTBC genetic variation, the efficacy of an HDT targeting a specific pathway might differ in specific populations. For example, a combination of zileuton and prostaglandin E2 (PGE2) as an HDT, which abrogates the detrimental type 1 interferon response and boosts eicosanoid levels, may find utility in TB therapy 95. The exonic g.760A allele in *ALOX5*, a key mediator of the type 1 IFN response to TB infection, is associated with protection against TB caused by *M. africanum* strains but not Mtb *senstu stricto* 96 (Table 2). While the functional implications of this variant are not known, the association could influence individual responses to the aforementioned HDT. In another example, the virulent lineage 2 HN878 isolate was shown to stimulate a high level of type 1 Interferon signaling in mice 18, 97, suggesting that higher levels of HDT that modulate eicosanoids might be required to be effective against infection with lineage 2 strains, or conversely that the HDT may actually be more effective in persons infected with lineage 2 strains.

## The impact of HIV co‐infection on immunological outcomes associated with different MTBC lineages

Due to dysregulated host immunity, it has been suggested that HIV‐1 co‐infection may facilitate the survival of less “fit” MTBC lineages within a population 2. It has been observed that TB is not transmitted efficiently from HIV‐1 co‐infected individuals, thus transmission predominantly occurs from HIV‐1 uninfected individuals to HIV‐1 co‐infected individuals 98, 99. A recent study modelling the impact of HIV‐1 on a large MDR outbreak in South America found no evidence of association between HIV‐1 and the mutation rate of Mtb, or MDR‐TB transmission 100.

MTBC creates an inflammatory milieu that exacerbates HIV‐1 infection and increases viral replication 26. Activation of nuclear factor (NF)‐κβ pathway and positive transcription elongation factor (P‐TEFβ), loss of CCAAT/enhancer‐binding protein and other chemokine and cytokine dependent pathways underlie this phenomenon 26. Clinical isolates of MTBC have been shown to differentially affect the replication of HIV‐1 in PBMC 101. For example, the lineage 4 CDC1551 strain induced higher levels of viral replication in human PBMCs than the lineage 2 HN878 strain 101. The HN878 strain contains an intact *pks1‐15* gene cluster resulting in production of a virulence associated phenolic glycolipid 20. Viral replication in PBMC co‐infected with a mutant HN878 *pks1‐15* deletion strain, which no longer produces the phenolic glycolipid, was similar to levels observed in PBMC co‐infected with the CDC1551 strain. This suggests that strain specific effects on HIV‐1 replication are associated with cell wall components of MTBC 101. MTBC dependent enhancement of HIV‐1 replication was subsequently shown to be a result of direct binding of transcription factor nuclear factor of activated T cells 5 (NFAT5) to the HIV‐1 LTR promoter 102. NFAT5 expression was induced in macrophages after MTBC infection in THP‐1 cells in a TLR2 dependent manner. While the authors did not examine the influence impact of MTBC strain variation in this study 102, the discussion above highlights the differential effects MTBC might have on TLRs. Together these findings suggest that MTBC strain variation may have important implications for of HIV‐1 replication, and disease progression during HIV/TB co‐infection 102.

## Conclusion

The studies discussed above indicate that genetic diversity within the MTBC can influence the host response to infection and during TB disease. However, the use of in vitro systems and animal models that do not completely recapitulate human TB makes it difficult to assess whether immunological variation associated with MTBC genetic diversity appreciably impacts clinical outcome, or whether TB control approaches should be tailored according to the infecting MTBC strain. The use of techniques such 2‐deoxy‐2‐[^18^F]‐fluoro‐D‐glucose (FDG) positron emission tomography/computed tomography (PET/CT) 103 and transcriptional profiling 54 during TB infection have recently provided important insights into pathophysiology within the human host and could be applied to investigate the impact of MTBC strain diversity on clinical outcome.

In addition to technical considerations associated with the immunological readout, many studies have characterized only a few representatives of a lineage, in many cases those that have been associated with well‐known outbreaks. Significant intra‐lineage diversity exists 12, 13 making extrapolation of phenotypes phenotypes associated with a few representatives to an entire lineage difficult. Moreover, as previously discussed, almost all MTBC WGS data have thus far been generated via short read sequencing techniques. Sequence in highly repetitive regions cannot reliably be determined using these techniques and are usually removed from downstream analysis 72. Short read data are also not useful for detecting genomic changes such as deletions and duplications, particularly those that span larger regions of the genome. These changes in genetic architecture, as well as epigenetic changes such as methylation patterns, may have important implications for MTBC physiology and associated host–pathogen interactions 104. Newer technologies, such as the PacBio system and hybrid sequencing strategies, which combine short‐read sequencing with single molecule sequencing 105, will provide the opportunity to investigate these genomic alterations and their associated immunological phenotypes.

In terms of childhood TB, studies suggest that associations between particular MTBC genotypes and TB in children is likely to reflect ongoing transmission from adults 106, 107, 108. However, variation in immunological outcomes due to MTBC genetic diversity in childhood TB specifically has not been extensively investigated. Given the immunobiology of TB disease in children is distinct from that of adults and varies with age 109, this presents an important area for further research.

Finally, definitively establishing genotype–phenotype relationships may benefit from allelic exchange type experiments that facilitate an understanding of cause and effect between a polymorphism and a particular phenotype 110. However, genetic manipulation of clinical isolates is likely to be challenging and complex phenotypes may result from epistatic interactions between multiple SNPs.

The aforementioned caveats notwithstanding, the studies discussed here have uncovered important variation in innate and adaptive immune responses to different MTBC strains, that warrants further investigation. The use of new immunological, clinical, and genomics techniques provides the opportunity to pursue and throw greater light on their significant in tuberculosis response.

## Conflict of interest

The authors declare no financial or commercial conflict of interest.

## References

[1] Brosch, R. , Gordon, S. V. , Marmiesse, M. , Brodin, P. , Buchrieser, C. , Eiglmeier, K. , Garnier, T. et al., A new evolutionary scenario for the *Mycobacterium tuberculosis* complex. Proc. Natl. Acad. Sci. U.S.A. 2002 99: 3684–3689. 11891304 10.1073/pnas.052548299 PMC122584

[2] Brites, D. and Gagneux, S. , Old and new selective pressures on *Mycobacterium tuberculosis* . Infect. Genet. Evol. 2012 12: 678–685. 21867778 10.1016/j.meegid.2011.08.010 PMC3253320

[3] Clifton‐Hadley, R. S. , Wilesmith, J. W. , Richards, M. S. , Upton, P. and Johnston, S. , The occurrence of *Mycobacterium bovis* infection in cattle in and around an area subject to extensive badger (*Meles meles*) control. Epidemiol. Infect. 1995 114: 179–193. 7867737 10.1017/s0950268800052031 PMC2271337

[4] Smith, N. H. , Crawshaw, T. , Parry, J. and Birtles, R. J. , *Mycobacterium microti*: more diverse than previously thought. J. Clin. Microbiol. 2009 47: 2551–2559. 19535520 10.1128/JCM.00638-09 PMC2725668

[5] Kiers, A. , Klarenbeek, A. , Mendelts, B. , van Soolingen, D. and Koëter, G. , Transmission of *Mycobacterium pinnipedii* to humans in a zoo with marine mammals. Int. J. Tuberc. Lung Dis. 2008 12: 1469–1473. 19017459

[6] Schoepf, K. , Prodinger, W. M. , Glawischnig, W. , Hofer, E. , Revilla‐Fernandez, S. , Hofrichter, J. , Fritz, J. et al., A two‐years' survey on the prevalence of tuberculosis caused by *Mycobacterium caprae* in Red Deer (*Cervus elaphus*) in the Tyrol, Austria. ISRN Vet Sci. 2012 2012: 245138–7. 23762580 10.5402/2012/245138 PMC3671721

[7] Gey van Pittius, N. C. , van Helden, P. D. and Warren, R. M. , Characterization of *Mycobacterium orygis* . Emerging Infect. Dis. 2012 18: 1708–1709. 23017199 10.3201/eid1810.120569 PMC3471637

[8] Alexander, K. A. , Laver, P. N. , Michel, A. L. , Williams, M. , van Helden, P. D. , Warren, R. M. and Gey van Pittius, N. C. , Novel *Mycobacterium tuberculosis* complex pathogen, M. mungi. Emerging Infect. Dis. 2010 16: 1296–1299. 10.3201/eid1608.100314 PMC3298296 20678329

[9] Gagneux, S. , Deriemer, K. , Van, T. , Kato‐Maeda, M. , de Jong, B. C. , Narayanan, S. , Nicol, M. et al., Variable host‐pathogen compatibility in *Mycobacterium tuberculosis* . Proc. Natl. Acad. Sci. U.S.A. 2006 103: 2869–2873. 16477032 10.1073/pnas.0511240103 PMC1413851

[10] Comas, I. , Chakravartti, J. , Small, P. M. , Galagan, J. , Niemann, S. , Kremer, K. , Ernst, J. D. et al., Human T cell epitopes of *Mycobacterium tuberculosis* are evolutionarily hyperconserved. Nat. Genet. 2010 42: 498–503. 20495566 10.1038/ng.590 PMC2883744

[11] de Jong, B. C. , Adetifa, I. , Walther, B. , Hill, P. C. , Antonio, M. , Ota, M. and Adegbola, R. A. , Differences between tuberculosis cases infected with *Mycobacterium africanum*, West African type 2, relative to Euro‐American *Mycobacterium tuberculosis*: an update. FEMS Immunol. Med. Microbiol. 2010 58: 102–105. 20002176 10.1111/j.1574-695X.2009.00628.x PMC2922882

[12] Merker, M. , Blin, C. , Mona, S. , Duforet‐Frebourg, N. , Lecher, S. , Willery, E. , Blum, M. G. B. et al., Evolutionary history and global spread of the *Mycobacterium tuberculosis* Beijing lineage. Nat. Genet. 2015 47: 242–249. 25599400 10.1038/ng.3195 PMC11044984

[13] Stucki, D. , Brites, D. , Jeljeli, L. , Coscolla, M. , Liu, Q. , Trauner, A. , Fenner, L. et al., *Mycobacterium tuberculosis* lineage 4 comprises globally distributed and geographically restricted sublineages. Nat. Genet. 2016 48: 1535–1543. 27798628 10.1038/ng.3704 PMC5238942

[14] Mitchison, D. A. , Wallace, J. G. , Bhatia, A. L. , Selkon, J. B. , Subbaiah, T. V. and Lancaster, M. C. , A comparison of the virulence in guinea‐pigs of South Indian and British tubercle bacilli. Tubercle. 1960 41: 1–22. 14423002 10.1016/s0041-3879(60)80019-0

[15] Nicol, M. P. and Wilkinson, R. J. , The clinical consequences of strain diversity in *Mycobacterium tuberculosis* . Trans. R. Soc. Trop. Med. Hyg. 2008 102: 955–965. 18513773 10.1016/j.trstmh.2008.03.025

[16] Valway, S. E. , Sanchez, M. P. , Shinnick, T. F. , Orme, I. , Agerton, T. , Hoy, D. , Jones, J. S. et al., An outbreak involving extensive transmission of a virulent strain of *Mycobacterium tuberculosis* . N. Engl. J. Med. 1998 338: 633–639. 9486991 10.1056/NEJM199803053381001

[17] Manca, C. , Tsenova, L. , Barry, C. E. , Bergtold, A. , Freeman, S. , Haslett, P. A. , Musser, J. M. et al., *Mycobacterium tuberculosis* CDC1551 induces a more vigorous host response in vivo and in vitro, but is not more virulent than other clinical isolates. J. Immunol. 1999 162: 6740–6746. 10352293

[18] Manca, C. , Tsenova, L. , Bergtold, A. , Freeman, S. , Tovey, M. , Musser, J. M. , Barry, C. E. et al., Virulence of a *Mycobacterium tuberculosis* clinical isolate in mice is determined by failure to induce Th1 type immunity and is associated with induction of IFN‐alpha /beta. Proc. Natl. Acad. Sci. U.S.A. 2001 98: 5752–5757. 11320211 10.1073/pnas.091096998 PMC33285

[19] Tsenova, L. , Ellison, E. , Harbacheuski, R. , Moreira, A. L. , Kurepina, N. , Reed, M. B. , Mathema, B. et al., Virulence of selected *Mycobacterium tuberculosis* clinical isolates in the rabbit model of meningitis is dependent on phenolic glycolipid produced by the bacilli. J. Infect. Dis. 2005 192: 98–106. 15942899 10.1086/430614

[20] Reed, M. B. , Domenech, P. , Manca, C. , Su, H. , Barczak, A. K. , Kreiswirth, B. N. , Kaplan, G. et al., A glycolipid of hypervirulent tuberculosis strains that inhibits the innate immune response. Nature 2004 431: 84–87. 15343336 10.1038/nature02837

[21] Click, E. S. , Moonan, P. K. , Winston, C. A. , Cowan, L. S. and Oeltmann, J. E. , Relationship between *Mycobacterium tuberculosis* phylogenetic lineage and clinical site of tuberculosis. Clin. Infect. Dis. 2012 54: 211–219. 22198989 10.1093/cid/cir788

[22] Thwaites, G. , Caws, M. , Chau, T. T. H. , D'Sa, A. , Lan, N. T. N. , Huyen, M. N. T. , Gagneux, S. et al., Relationship between *Mycobacterium tuberculosis* genotype and the clinical phenotype of pulmonary and meningeal tuberculosis. J. Clin. Microbiol. 2008 46: 1363–1368. 18287322 10.1128/JCM.02180-07 PMC2292951

[23] Coscolla, M. and Gagneux, S. , Does *M. tuberculosis* genomic diversity explain disease diversity? *Drug Discov* . Today Dis. Mech. 2010 7: e43–e59. 10.1016/j.ddmec.2010.09.004 PMC2976975 21076640

[24] Stein, C. M. , Genetic epidemiology of tuberculosis susceptibility: impact of study design. PLoS Pathog. 2011 7: e1001189. 21283783 10.1371/journal.ppat.1001189 PMC3024264

[25] Stamm, C. E. , Collins, A. C. and Shiloh, M. U. , Sensing of *Mycobacterium tuberculosis* and consequences to both host and bacillus. Immunol. Rev. 2015 264: 204–219. 25703561 10.1111/imr.12263 PMC4339209

[26] O'Garra, A. , Redford, P. S. , McNab, F. W. , Bloom, C. I. , Wilkinson, R. J. and Berry, M. P. R. , The immune response in tuberculosis. Annu. Rev. Immunol. 2013 31: 475–527. 23516984 10.1146/annurev-immunol-032712-095939

[27] Kallenius, G. , Correia‐Neves, M. , Buteme, H. , Hamasur, B. and Svenson, S. B. , Lipoarabinomannan, and its related glycolipids, induce divergent and opposing immune responses to *Mycobacterium tuberculosis* depending on structural diversity and experimental variations. Tuberculosis (Edinb). 2016 96: 120–130. 26586646 10.1016/j.tube.2015.09.005

[28] Smet, M. , Pollard, C. , De Beuckelaer, A. , Van Hoecke, L. , Vander Beken, S. , De Koker, S. , Al Dulayymi, J. R. et al., *Mycobacterium tuberculosis*‐associated synthetic mycolates differentially exert immune stimulatory adjuvant activity. Eur. J. Immunol. 2016 46: 2149–2154. 27349218 10.1002/eji.201646357

[29] Kleinnijenhuis, J. , Oosting, M. , Joosten, L. A. B. , Netea, M. G. and van Crevel, R. , Innate immune recognition of *Mycobacterium tuberculosis* . Clin. Dev. Immunol. 2011 2011: 405310–405312. 21603213 10.1155/2011/405310 PMC3095423

[30] Tailleux, L. , Schwartz, O. , Herrmann, J.‐L. , Pivert, E. , Jackson, M. , Amara, A. , Legres, L. et al., DC‐SIGN is the major *Mycobacterium tuberculosis* receptor on human dendritic cells. J. Exp. Med. 2003 197: 121–127. 12515819 10.1084/jem.20021468 PMC2193794

[31] Schlesinger, L. S. , Kaufman, T. M. , Iyer, S. , Hull, S. R. and Marchiando, L. K. , Differences in mannose receptor‐mediated uptake of lipoarabinomannan from virulent and attenuated strains of *Mycobacterium tuberculosis* by human macrophages. J. Immunol. 1996 157: 4568–4575. 8906835

[32] Torrelles, J. B. , Knaup, R. , Kolareth, A. , Slepushkina, T. , Kaufman, T. M. , Kang, P. , Hill, P. J. et al., Identification of *Mycobacterium tuberculosis* clinical isolates with altered phagocytosis by human macrophages due to a truncated lipoarabinomannan. J. Biol. Chem. 2008 283: 31417–31428. 18784076 10.1074/jbc.M806350200 PMC2581576

[33] Carmona, J. , Cruz, A. , Moreira‐Teixeira, L. , Sousa, C. , Sousa, J. , Osório, N. S. , Saraiva, A. L. et al., *Mycobacterium tuberculosis* strains are differentially recognized by TLRs with an impact on the immune response. PLOS ONE 2013 8: e67277. 23840651 10.1371/journal.pone.0067277 PMC3693941

[34] van Crevel, R. , Ottenhoff, T. H. M. and van der Meer, J. W. M. , Innate immunity to *Mycobacterium tuberculosis* . Clin. Microbiol. Rev. 2002 15: 294–309. 11932234 10.1128/CMR.15.2.294-309.2002 PMC118070

[35] Rocha‐Ramírez, L. M. , Estrada‐García, I. , López‐Marín, L. M. , Segura‐Salinas, E. , Méndez‐Aragón, P. , van Soolingen, D. , Torres‐González, R. et al., *Mycobacterium tuberculosi*s lipids regulate cytokines, TLR‐2/4 and MHC class II expression in human macrophages. Tuberculosis 2008 88: 212–220. 18222732 10.1016/j.tube.2007.10.003

[36] Gopal, R. , Monin, L. , Slight, S. , Uche, U. , Blanchard, E. , Fallert Junecko, B. A. , Ramos‐Payan, R. et al., Unexpected role for IL‐17 in protective immunity against hypervirulent *Mycobacterium tuberculosis* HN878 infection. PLoS Pathog. 2014 10: e1004099. 24831696 10.1371/journal.ppat.1004099 PMC4022785

[37] Caws, M. , Thwaites, G. , Dunstan, S. , Hawn, T. R. , Lan, N. T. N. , Thuong, N. T. T. , Stepniewska, K. et al., The influence of host and bacterial genotype on the development of disseminated disease with *Mycobacterium tuberculosis* . PLoS Pathog. 2008 4: e1000034. 18369480 10.1371/journal.ppat.1000034 PMC2268004

[38] Ghiran, I. , Barbashov, S. F. , Klickstein, L. B. , Tas, S. W. , Jensenius, J. C. and Nicholson‐Weller, A. , Complement receptor 1/CD35 is a receptor for mannan‐binding lectin. J. Exp. Med. 2000 192: 1797–1808. 11120776 10.1084/jem.192.12.1797 PMC2213499

[39] Bonar, A. , Chmiela, M. , Rudnicka, W. and Rózalska, B. , Mannose‐binding lectin enhances the attachment and phagocytosis of mycobacteria *in vitro* . Arch. Immunol. Ther. Exp. (Warsz.). 2005 53: 437–441. 16314827

[40] Ezekowitz, R. A. , Role of the mannose‐binding lectin in innate immunity. J. Infect. Dis. 2003 187(Suppl 2): S335–S339. 12792848 10.1086/374746

[41] Singla, N. , Gupta, D. , Joshi, A. , Batra, N. , Singh, J. and Birbian, N. , Association of mannose‐binding lectin gene polymorphism with tuberculosis susceptibility and sputum conversion time. Int. J. Immunogenet. 2012 39: 10–14. 22050925 10.1111/j.1744-313X.2011.01047.x

[42] Thye, T. , Niemann, S. , Walter, K. , Homolka, S. , Intemann, C. D. , Chinbuah, M. A. , Enimil, A. et al., Variant G57E of mannose binding lectin associated with protection against tuberculosis caused by *Mycobacterium africanum* but not by *M. tuberculosis* . PLOS ONE 2011 6: e20908. 21695215 10.1371/journal.pone.0020908 PMC3112207

[43] Eisen, D. P. and Minchinton, R. M. , Impact of mannose‐binding lectin on susceptibility to infectious diseases. Clin. Infect. Dis. 2003 37: 1496–1505. 14614673 10.1086/379324

[44] Azad, A. K. , Sadee, W. and Schlesinger, L. S. , Innate immune gene polymorphisms in tuberculosis. Infect. Immun. 2012 80: 3343–3359. 22825450 10.1128/IAI.00443-12 PMC3457569

[45] Scriba, T. J. , Coussens, A. K. and Fletcher, H. A. , Human immunology of tuberculosis. Microbiol Spectr. 2016 4. 10.1128/microbiolspec.TBTB2-0016-2016 27726784

[46] Theus, S. A. , Cave, M. D. , Eisenach, K. , Walrath, J. , Lee, H. , Mackay, W. , Whalen, C. et al., Differences in the growth of paired Ugandan isolates of *Mycobacterium tuberculosis* within human mononuclear phagocytes correlate with epidemiological evidence of strain virulence. Infect. Immun. 2006 74: 6865–6876. 16982841 10.1128/IAI.00561-06 PMC1698107

[47] Portevin, D. , Gagneux, S. , Comas, I. and Young, D. , Human macrophage responses to clinical isolates from the *Mycobacterium tuberculosis* complex discriminate between ancient and modern lineages. PLoS Pathog. 2011 7: e1001307. 21408618 10.1371/journal.ppat.1001307 PMC3048359

[48] Ribeiro, S. C. M. , Gomes, L. L. , Amaral, E. P. , Andrade, M. R. M. , Almeida, F. M. , Rezende, A. L. , Lanes, V. R. et al., *Mycobacterium tuberculosis* strains of the modern sublineage of the Beijing family are more likely to display increased virulence than strains of the ancient sublineage. J. Clin. Microbiol. 2014 52: 2615–2624. 24829250 10.1128/JCM.00498-14 PMC4097719

[49] Homolka, S. , Niemann, S. , Russell, D. G. and Rohde, K. H. , Functional genetic diversity among *Mycobacterium tuberculosis* complex clinical isolates: delineation of conserved core and lineage‐specific transcriptomes during intracellular survival. PLoS Pathog. 2010 6: e1000988. 20628579 10.1371/journal.ppat.1000988 PMC2900310

[50] Sarkar, R. , Lenders, L. , Wilkinson, K. A. , Wilkinson, R. J. and Nicol, M. P. , Modern lineages of *Mycobacterium tuberculosis* exhibit lineage‐specific patterns of growth and cytokine induction in human monocyte‐derived macrophages. PLOS ONE 2012 7: e43170. 22916219 10.1371/journal.pone.0043170 PMC3420893

[51] Manca, C. , Reed, M. B. , Freeman, S. , Mathema, B. , Kreiswirth, B. , Barry, C. E. and Kaplan, G. , Differential monocyte activation underlies strain‐specific *Mycobacterium tuberculosis* pathogenesis. Infect. Immun. 2004 72: 5511–5514. 15322056 10.1128/IAI.72.9.5511-5514.2004 PMC517425

[52] Newton, S. M. , Smith, R. J. , Wilkinson, K. A. , Nicol, M. P. , Garton, N. J. , Staples, K. J. , Stewart, G. R. et al., A deletion defining a common Asian lineage of *Mycobacterium tuberculosis* associates with immune subversion. Proc. Natl. Acad. Sci. U.S.A. 2006 103: 15594–15598. 17028173 10.1073/pnas.0604283103 PMC1622867

[53] Eum, S. Y. , Kong, J.‐H. , Hong, M.‐S. , Lee, Y.‐J. , Kim, J. H. , Hwang, S.‐H. , Cho, S.‐N. et al., Neutrophils are the predominant infected phagocytic cells in the airways of patients with active pulmonary TB. Chest 2010 137: 122–128. 19749004 10.1378/chest.09-0903 PMC2803122

[54] Berry, M. P. R. , Graham, C. M. , McNab, F. W. , Xu, Z. , Bloch, S. A. A. , Oni, T. , Wilkinson, K. A. et al., An interferon‐inducible neutrophil‐driven blood transcriptional signature in human tuberculosis. Nature 2010 466: 973–977. 20725040 10.1038/nature09247 PMC3492754

[55] Martineau, A. R. , Newton, S. M. , Wilkinson, K. A. , Kampmann, B. , Hall, B. M. , Nawroly, N. , Packe, G. E. et al., Neutrophil‐mediated innate immune resistance to mycobacteria. J. Clin. Invest. 2007 117: 1988–1994. 17607367 10.1172/JCI31097 PMC1904316

[56] Kisich, K. O. , Higgins, M. , Diamond, G. and Heifets, L. , Tumor necrosis factor alpha stimulates killing of *Mycobacterium tuberculosis* by human neutrophils. Infect. Immun. 2002 70: 4591–4599. 12117972 10.1128/IAI.70.8.4591-4599.2002 PMC128192

[57] Romero, M. M. , Basile, J. I. , Lopez, B. , Ritacco, V. , Barrera, L. , Sasiain, M. D. C. and Alemán, M. , Outbreaks of *Mycobacterium tuberculosis* MDR strains differentially induce neutrophil respiratory burst involving lipid rafts, p38 MAPK and Syk. BMC Infect. Dis. 2014 14: 262. 24886274 10.1186/1471-2334-14-262 PMC4049492

[58] Romero, M. M. , Balboa, L. , Basile, J. I. , Lopez, B. , Ritacco, V. , la Barrera de, S. S. , Sasiain, M. C. et al., Clinical isolates of *Mycobacterium tuberculosis* differ in their ability to induce respiratory burst and apoptosis in neutrophils as a possible mechanism of immune escape. Clin. Dev. Immunol. 2012 2012: 152546–11. 22778761 10.1155/2012/152546 PMC3388301

[59] Reyes‐Martínez, J. E. , Nieto‐Patlán, E. , Nieto‐Patlán, A. , Gonzaga‐Bernachi, J. , Santos‐Mendoza, T. , Serafín‐López, J. , Chávez‐Blanco, A. et al., Differential activation of dendritic cells by *Mycobacterium tuberculosis* Beijing genotype. Immunol. Invest. 2014 43: 436–446. 24654560 10.3109/08820139.2014.880120

[60] Rajashree, P. , Supriya, P. and Das, S. D. , Differential migration of human monocyte‐derived dendritic cells after infection with prevalent clinical strains of *Mycobacterium tuberculosis* . Immunobiology 2008 213: 567–575. 18656704 10.1016/j.imbio.2008.01.007

[61] Wang, C. , Peyron, P. , Mestre, O. , Kaplan, G. , van Soolingen, D. , Gao, Q. , Gicquel, B. et al., Innate immune response to *Mycobacterium tuberculosis* Beijing and other genotypes. PLOS ONE 2010 5: e13594. 21049036 10.1371/journal.pone.0013594 PMC2963601

[62] Krishnan, N. , Malaga, W. , Constant, P. , Caws, M. , Tran, T. H. C. , Salmons, J. , Nguyen, T. N. L. et al., *Mycobacterium tuberculosis* lineage influences innate immune response and virulence and is associated with distinct cell envelope lipid profiles. PLOS ONE 2011 6: e23870. 21931620 10.1371/journal.pone.0023870 PMC3169546

[63] Kaufmann, S. H. , How can immunology contribute to the control of tuberculosis? Nat. Rev. Immunol. 2001 1: 20–30. 11905811 10.1038/35095558

[64] Canaday, D. H. , Wilkinson, R. J. , Li, Q. , Harding, C. V. , Silver, R. F. and Boom, W. H. , CD4(+) and CD8(+) T cells kill intracellular *Mycobacterium tuberculosis* by a perforin and Fas/Fas ligand‐independent mechanism. J. Immunol. 2001 167: 2734–2742. 11509617 10.4049/jimmunol.167.5.2734

[65] Tientcheu, L. D. , Sutherland, J. S. , de Jong, B. C. , Kampmann, B. , Jafali, J. , Adetifa, I. M. , Antonio, M. et al., Differences in T‐cell responses between *Mycobacterium tuberculosis* and *Mycobacterium africanum*‐infected patients. Eur. J. Immunol. 2014 44: 1387–1398. 24481948 10.1002/eji.201343956

[66] Zimmermann, N. , Thormann, V. , Hu, B. , Köhler, A.‐B. , Imai‐Matsushima, A. , Locht, C. , Arnett, E. et al., Human isotype‐dependent inhibitory antibody responses against *Mycobacterium tuberculosis* . EMBO Mol. Med. 2016: e201606330. 10.15252/emmm.201606330 PMC5090662 27729388

[67] Lu, L. L. , Chung, A. W. , Rosebrock, T. R. , Ghebremichael, M. , Yu, W. H. , Grace, P. S. , Schoen, M. K. et al., A functional role for antibodies in Tuberculosis. Cell. 2016 167: 433–443.e14. 27667685 10.1016/j.cell.2016.08.072 PMC5526202

[68] Kato‐Maeda, M. , Shanley, C. A. , Ackart, D. , Jarlsberg, L. G. , Shang, S. , Obregon‐Henao, A. , Harton, M. et al., Beijing sublineages of *Mycobacterium tuberculosis* differ in pathogenicity in the guinea pig. Clin. Vaccine Immunol. 2012 19: 1227–1237. 22718126 10.1128/CVI.00250-12 PMC3416080

[69] Kim, W. S. , Kim, J.‐S. , Cha, S. B. , Han, S. J. , Kim, H. , Kwon, K. W. , Kim, S. J. et al., Virulence‐Dependent alterations in the kinetics of immune cells during pulmonary infection by *Mycobacterium tuberculosis* . PLOS ONE 2015 10: e0145234. 26675186 10.1371/journal.pone.0145234 PMC4682951

[70] Ordway, D. , Henao‐Tamayo, M. , Harton, M. , Palanisamy, G. , Troudt, J. , Shanley, C. , Basaraba, R. J. et al., The hypervirulent *Mycobacterium tuberculosis* strain HN878 induces a potent TH1 response followed by rapid down‐regulation. J. Immunol. 2007 179: 522–531. 17579073 10.4049/jimmunol.179.1.522

[71] Copin, R. , Coscollá, M. , Seiffert, S. N. and Bothamley, G. , Sequence diversity in the *pe_pgrs* genes of *Mycobacterium tuberculosis* is independent of human T cell recognition. MBio. 2014 5: e00960‐13. 24425732 10.1128/mBio.00960-13 PMC3903279

[72] Bryant, J. M. , Harris, S. R. , Parkhill, J. , Dawson, R. , Diacon, A. H. , van Helden, P. , Pym, A. et al., Whole‐genome sequencing to establish relapse or re‐infection with *Mycobacterium tuberculosis*: a retrospective observational study. Lancet Respir. Med. 2013 1: 786–792. 24461758 10.1016/S2213-2600(13)70231-5 PMC3861685

[73] Sampson, S. L. , Mycobacterial PE/PPE proteins at the host‐pathogen interface. Clin. Dev. Immunol. 2011 2011: 497203–497211. 21318182 10.1155/2011/497203 PMC3034920

[74] Vordermeier, H. M. , Hewinson, R. G. , Wilkinson, R. J. , Wilkinson, K. A. , Gideon, H. P. , Young, D. B. and Sampson, S. L. , Conserved immune recognition hierarchy of mycobacterial PE/PPE proteins during infection in natural hosts. PLOS ONE 2012 7: e40890. 22870206 10.1371/journal.pone.0040890 PMC3411574

[75] Coscolla, M. , Copin, R. , Sutherland, J. , Gehre, F. , de Jong, B. , Owolabi, O. , Mbayo, G. et al., *M. tuberculosis* T Cell epitope analysis reveals paucity of antigenic variation and identifies rare variable TB antigens. Cell Host & Microbe. 2015 18: 538–548. 26607161 10.1016/j.chom.2015.10.008 PMC4758912

[76] Parwati, I. , van Crevel, R. and van Soolingen, D. , Possible underlying mechanisms for successful emergence of the *Mycobacterium tuberculosis* Beijing genotype strains. The Lancet Infect. Dis. 2010 10: 103–111. 20113979 10.1016/S1473-3099(09)70330-5

[77] Abebe, F. and Bjune, G. , The emergence of Beijing family genotypes of *Mycobacterium tuberculosis* and low‐level protection by bacille Calmette‐Guérin (BCG) vaccines: is there a link? Clin. Exp. Immunol. 2006 145: 389–397. 16907905 10.1111/j.1365-2249.2006.03162.x PMC1809707

[78] López, B. , Aguilar, D. , Orozco, H. , Burger, M. , Espitia, C. , Ritacco, V. , Barrera, L. et al., A marked difference in pathogenesis and immune response induced by different *Mycobacterium tuberculosis* genotypes. Clin. Exp. Immunol. 2003 133: 30–37. 12823275 10.1046/j.1365-2249.2003.02171.x PMC1808750

[79] Tsenova, L. , Harbacheuski, R. , Sung, N. , Ellison, E. , Fallows, D. and Kaplan, G. , BCG vaccination confers poor protection against *M. tuberculosis* HN878‐induced central nervous system disease. Vaccine. 2007 25: 5126–5132. 17241704 10.1016/j.vaccine.2006.11.024 PMC1994581

[80] Jeon, B. Y. , Derrick, S. C. , Lim, J. , Kolibab, K. , Dheenadhayalan, V. , Yang, A. L. , Kreiswirth, B. et al., *Mycobacterium bovis* BCG immunization induces protective immunity against nine different *Mycobacterium tuberculosis* strains in mice. Infect. Immun. 2008 76: 5173–5180. 18710860 10.1128/IAI.00019-08 PMC2573355

[81] Henao‐Tamayo, M. , Shanley, C. A. , Verma, D. , Zilavy, A. , Stapleton, M. C. , Furney, S. K. , Podell, B. et al., The efficacy of the BCG vaccine against newly emerging clinical strains of *Mycobacterium tuberculosis* . PLOS ONE 2015 10: e0136500. 26368806 10.1371/journal.pone.0136500 PMC4569086

[82] Grode, L. , Seiler, P. , Baumann, S. , Hess, J. , Brinkmann, V. , Nasser Eddine, A. , Mann, P. et al., Increased vaccine efficacy against tuberculosis of recombinant *Mycobacterium bovis* bacille Calmette‐Guérin mutants that secrete listeriolysin. J. Clin. Invest. 2005 115: 2472–2479. 16110326 10.1172/JCI24617 PMC1187936

[83] Fletcher, H. A. and Schrager, L. , TB vaccine development and the End TB Strategy: importance and current status. Trans. R. Soc. Trop. Med. Hyg. 2016 110: 212–218. 27076508 10.1093/trstmh/trw016 PMC4830404

[84] Coscolla, M. and Gagneux, S. , Consequences of genomic diversity in *Mycobacterium tuberculosis* . Semin. Immunol. 2014 26: 431–444. 25453224 10.1016/j.smim.2014.09.012 PMC4314449

[85] Gillard, P. , Yang, P.‐C. , Danilovits, M. , Su, W.‐J. , Cheng, S.‐L. , Pehme, L. , Bollaerts, A. et al., Safety and immunogenicity of the M72/AS01E candidate tuberculosis vaccine in adults with tuberculosis: a phase II randomised study. Tuberculosis (Edinb). 2016 100: 118–127. 27553419 10.1016/j.tube.2016.07.005

[86] Homolka, S. , Ubben, T. and Niemann, S. , High sequence variability of the *ppE18* gene of clinical *Mycobacterium tuberculosis* complex strains potentially impacts effectivity of vaccine candidate M72/AS01E. PLOS ONE 2016 11: e0152200. 27011018 10.1371/journal.pone.0152200 PMC4806982

[87] Hebert, A. M. , Talarico, S. , Yang, D. , Durmaz, R. , Marrs, C. F. , Zhang, L. , Foxman, B. et al., DNA polymorphisms in the *pepA* and *PPE18* genes among clinical strains of *Mycobacterium tuberculosis*: implications for vaccine efficacy. Infect. Immun. 2007 75: 5798–5805. 17893137 10.1128/IAI.00335-07 PMC2168324

[88] Anderson, S. T. , Williams, A. J. , Brown, J. R. , Newton, S. M. , Simsova, M. , Nicol, M. P. , Sebo, P. et al., Transmission of *Mycobacterium tuberculosis* undetected by tuberculin skin testing. Am. J. Respir. Crit. Care Med. 2006 173: 1038–1042. 16456140 10.1164/rccm.200509-1526OC

[89] Pai, M. , Behr, M. A. , Dowdy, D. , Dheda, K. , Divangahi, M. , Boehme, C. C. , Ginsberg, A. et al., Tuberculosis. Nat. Rev. Dis. Primers. 2016 2: 16076. 27784885 10.1038/nrdp.2016.76

[90] de Jong, B. C. , Hill, P. C. , Brookes, R. H. , Gagneux, S. , Jeffries, D. J. , Otu, J. K. , Donkor, S. A. et al., *Mycobacterium africanum* elicits an attenuated T cell response to early secreted antigenic target, 6 kDa, in patients with tuberculosis and their household contacts. J. Infect. Dis. 2006 193: 1279–1286. 16586366 10.1086/502977

[91] Adekambi, T. , Ibegbu, C. C. , Cagle, S. , Kalokhe, A. S. , Wang, Y. F. , Hu, Y. , Day, C. L. et al., Biomarkers on patient T cells diagnose active tuberculosis and monitor treatment response. J. Clin. Invest. 2015 125: 3723–3723. 10.1172/JCI83279 PMC4588276 26325038

[92] Riou, C. , Perez Peixoto, B. , Roberts, L. , Ronacher, K. , Walzl, G. , Manca, C. , Rustomjee, R. et al., Effect of standard tuberculosis treatment on plasma cytokine levels in patients with active pulmonary tuberculosis. PLOS ONE 2012 7: e36886. 22606304 10.1371/journal.pone.0036886 PMC3351475

[93] Tientcheu, L. D. , Haks, M. C. , Agbla, S. C. , Sutherland, J. S. , Adetifa, I. M. , Donkor, S. , Quinten, E. et al., Host immune responses differ between *M. africanum*‐ and *M. tuberculosis*‐infected patients following standard anti‐tuberculosis treatment. PLoS Negl. Trop. Dis. 2016 10: e0004701. 27192147 10.1371/journal.pntd.0004701 PMC4871581

[94] Zumla, A. , Maeurer, M. , Host‐Directed Therapies Network , Chakaya, J. , Hoelscher, M. , Ntoumi, F. , Rustomjee, R. et al., Towards host‐directed therapies for tuberculosis. Nat. Rev. Drug Discov. 2015 14: 511–512. 26184493 10.1038/nrd4696

[95] Mayer‐Barber, K. D. , Andrade, B. B. , Oland, S. D. , Amaral, E. P. , Barber, D. L. , Gonzales, J. , Derrick, S. C. et al., Host‐directed therapy of tuberculosis based on interleukin‐1 and type I interferon crosstalk. Nature 2014 511: 99–103. 24990750 10.1038/nature13489 PMC4809146

[96] Herb, F. , Thye, T. , Niemann, S. , Browne, E. N. L. , Chinbuah, M. A. , Gyapong, J. , Osei, I. et al., ALOX5 variants associated with susceptibility to human pulmonary tuberculosis. Hum. Mol. Genet. 2008 17: 1052–1060. 18174194 10.1093/hmg/ddm378

[97] Manca, C. , Tsenova, L. , Freeman, S. , Barczak, A. K. , Tovey, M. , Murray, P. J. , Barry, C. et al., Hypervirulent *M. tuberculosis* W/Beijing strains upregulate type I IFNs and increase expression of negative regulators of the Jak‐Stat pathway. J. Interferon Cytokine Res. 2005 25: 694–701. 16318583 10.1089/jir.2005.25.694

[98] Guerra‐Assunção, J. A. , Crampin, A. C. , Houben, R. M. G. J. , Mzembe, T. , Mallard, K. , Coll, F. , Khan, P. et al., Large‐scale whole genome sequencing of *M. tuberculosis* provides insights into transmission in a high prevalence area. eLife Sciences 2015 4: 110. 10.7554/eLife.05166 PMC4384740 25732036

[99] Middelkoop, K. , Bekker, L.‐G. , Mathema, B. , Shashkina, E. , Kurepina, N. , Whitelaw, A. , Fallows, D. et al., Molecular epidemiology of *Mycobacterium tuberculosis* in a South African community with high HIV prevalence. J. Infect. Dis. 2009 200: 1207–1211. 19764885 10.1086/605930 PMC2932637

[100] Eldholm, V. , Rieux, A. , Monteserin, J. , Lopez, J. M. , Palmero, D. , Lopez, B. , Ritacco, V. et al., Impact of HIV co‐infection on the evolution and transmission of multidrug‐resistant tuberculosis. eLife Sciences 2016 5: 306. 10.7554/eLife.16644 PMC4978521 27502557

[101] Ranjbar, S. , Boshoff, H. I. , Mulder, A. , Siddiqi, N. , Rubin, E. J. and Goldfeld, A. E. , HIV‐1 replication is differentially regulated by distinct clinical strains of *Mycobacterium tuberculosis* . PLOS ONE 2009 4: e6116. 19568431 10.1371/journal.pone.0006116 PMC2699470

[102] Ranjbar, S. , asenosky, L. D. , Chow, N. and Goldfeld, A. E. , Regulation of *Mycobacterium tuberculosis*‐dependent HIV‐1 transcription reveals a new role for NFAT5 in the toll‐like receptor pathway. PLoS Pathog 2012 8: e1002620. 22496647 10.1371/journal.ppat.1002620 PMC3320587

[103] Chen, R. Y. , Dodd, L. E. , Lee, M. , Paripati, P. , Hammoud, D. A. , Mountz, J. M. , Jeon, D. et al., PET/CT imaging correlates with treatment outcome in patients with multidrug‐resistant tuberculosis. Sci. Transl. Med. 2014 6: 265ra166–265ra166. 10.1126/scitranslmed.3009501 PMC5567784 25473034

[104] Domenech, P. , Rog, A. , Moolji, J.‐U.‐D. , Radomski, N. , Fallow, A. , Leon‐Solis, L. , Bowes, J. et al., Origins of a 350‐kilobase genomic duplication in *Mycobacterium tuberculosis* and its impact on virulence. Infect. Immun. 2014 82: 2902–2912. 24778110 10.1128/IAI.01791-14 PMC4097636

[105] Rhoads, A. and Au, K. F. , PacBio sequencing and its applications. Genomics Proteomics Bioinformatics 2015 13: 278–289. 26542840 10.1016/j.gpb.2015.08.002 PMC4678779

[106] Nicol, M. P. , Sola, C. , February, B. , Rastogi, N. , Steyn, L. and Wilkinson, R. J. , Distribution of strain families of *Mycobacterium tuberculosis* causing pulmonary and extrapulmonary disease in hospitalized children in Cape Town, South Africa. J. Clin. Microbiol. 2005 43: 5779–5781. 16272518 10.1128/JCM.43.11.5779-5781.2005 PMC1287833

[107] Cowley, D. , Govender, D. , February, B. , Wolfe, M. , Steyn, L. , Evans, J. , Wilkinson, R. J. et al., Recent and rapid emergence of W‐Beijing strains of *Mycobacterium tuberculosis* in Cape Town, South Africa. Clin. Infect. Dis. 2008 47: 1252–1259. 18834315 10.1086/592575

[108] Wampande, E. M. , Mupere, E. , Jaganath, D. , Nsereko, M. , Mayanja, H. K. , Eisenach, K. , Boom, W. H. et al., Distribution and transmission of *Mycobacterium tuberculosis* complex lineages among children in peri‐urban Kampala, Uganda. BMC Pediatr. 2015 15: 140. 26424324 10.1186/s12887-015-0455-z PMC4588907

[109] Jones, C. , Whittaker, E. , Bamford, A. and Kampmann, B. , Immunology and pathogenesis of childhood TB. Paediatr. Respir. Rev. 2011 12: 3–8. 21172668 10.1016/j.prrv.2010.09.006

[110] Warner, D. F. , Koch, A. and Mizrahi, V. , Diversity and disease pathogenesis in *Mycobacterium tuberculosis* . Trends Microbiol. 2015 23: 14–21. 25468790 10.1016/j.tim.2014.10.005

[111] Galagan, J. E. , Genomic insights into tuberculosis. Nat. Rev. Genet. 2014 15: 307–320. 24662221 10.1038/nrg3664

[112] Hershberg, R. , Lipatov, M. , Small, P. M. , Sheffer, H. , Niemann, S. , Homolka, S. , Roach, J. C. et al., High functional diversity in *Mycobacterium tuberculosis* driven by genetic drift and human demography. PLOS Biol. 2008 6: e311. 19090620 10.1371/journal.pbio.0060311 PMC2602723

[113] Comas, I. , Homolka, S. , Niemann, S. and Gagneux, S. , Genotyping of genetically monomorphic bacteria: DNA sequencing in *Mycobacterium tuberculosis* highlights the limitations of current methodologies. PLOS ONE 2009 4: e7815. 19915672 10.1371/journal.pone.0007815 PMC2772813

[114] Kato‐Maeda, M. , Metcalfe, J. Z. and Flores, L. , Genotyping of *Mycobacterium tuberculosis*: application in epidemiologic studies. Future Microbiol. 2011 6: 203–216. 21366420 10.2217/fmb.10.165 PMC4296029

[115] Loman, N. J. and Pallen, M. J. , Twenty years of bacterial genome sequencing. Nat. Rev. Microbiol. 2015 13: 787–794. 26548914 10.1038/nrmicro3565

[116] Intemann, C. D. , Thye, T. , Niemann, S. , Browne, E. N. L. , Amanua Chinbuah, M. , Enimil, A. , Gyapong, J. et al., Autophagy gene variant IRGM ‐261T contributes to protection from tuberculosis caused by *Mycobacterium tuberculosis* but not by *M. africanum* strains. PLoS Pathog. 2009 5: e1000577. 19750224 10.1371/journal.ppat.1000577 PMC2735778

[117] Intemann, C. D. , Thye, T. , Förster, B. , Owusu‐Dabo, E. , Gyapong, J. , Horstmann, R. D. and Meyer, C. G. , MCP1 haplotypes associated with protection from pulmonary tuberculosis. BMC Genet. 2011 12: 34. 21504590 10.1186/1471-2156-12-34 PMC3107163

[118] Thye, T. , Nejentsev, S. , Intemann, C. D. , Browne, E. N. , Chinbuah, M. A. , Gyapong, J. , Osei, I. et al., MCP‐1 promoter variant ‐362C associated with protection from pulmonary tuberculosis in Ghana, West Africa. Hum. Mol. Genet. 2009 18: 381–388. 18940815 10.1093/hmg/ddn352 PMC2638774

[119] White, M. J. , Tacconelli, A. , Chen, J. S. , Wejse, C. , Hill, P. C. , Gomes, V. F. , Velez‐Edwards, D. R. et al., Epiregulin (EREG) and human V‐ATPase (TCIRG1): genetic variation, ethnicity and pulmonary tuberculosis susceptibility in Guinea‐Bissau and The Gambia. Genes Immun. 2014 15: 370–377. 24898387 10.1038/gene.2014.28 PMC5789787

[120] Salie, M. , an der Merwe, L. , Möller, M. , Daya, M. , van der Spuy, G. D. , van Helden, P. D. , Martin, M. P. et al., Associations between human leukocyte antigen class I variants and the *Mycobacterium tuberculosis* subtypes causing disease. J. Infect. Dis. 2014 209: 216–223. 23945374 10.1093/infdis/jit443 PMC3873786

[121] Thuong, N. T. T. , Tram, T. T. B. , Dinh, T. D. , Thai, P. V. K. , Heemskerk, D. , Bang, N. D. , Chau, T. T. H. et al., MARCO variants are associated with phagocytosis, pulmonary tuberculosis susceptibility and Beijing lineage. Genes Immun. 2016 17: 419–425. 27853145 10.1038/gene.2016.43 PMC5133378

[122] van Crevel, R. , Parwati, I. , Sahiratmadja, E. , Marzuki, S. , Ottenhoff, T. H. M. , Netea, M. G. , van der Ven, A. et al., Infection with *Mycobacterium tuberculosis* Beijing genotype strains is associated with polymorphisms in SLC11A1/NRAMP1 in Indonesian patients with tuberculosis. J Infect Dis. 2009 200: 1671–1674. 19863441 10.1086/648477

[123] Ogarkov, O. , Mokrousov, I. , Sinkov, V. , Zhdanova, S. , Antipina, S. and Savilov, E. , ‘Lethal’ combination of *Mycobacterium tuberculosis* Beijing genotype and human CD209 ‐336G allele in Russian male population. Infect. Genet. Evol. 2012 12: 732–736. 22027159 10.1016/j.meegid.2011.10.005

